# Target Salience and Search Modes: A Commentary on Theeuwes (2023)

**DOI:** 10.5334/joc.279

**Published:** 2023-07-06

**Authors:** Heinrich R. Liesefeld, Hermann J. Müller

**Affiliations:** 1 University of Bremen, Germany; 2 Ludwig-Maximilians-Universität München, Germany

**Keywords:** search modes, clump scanning, priority guidance, distraction, visual search, salience

## Abstract

In a healthy scientific community, theories influence each other and promising ideas are embraced by competing theoretical camps. We are therefore pleased that Theeuwes (2023) now agrees with core points of our theoretical position (Liesefeld et al., 2021; Liesefeld & Müller, 2020), most notably, the central role target salience plays for interference by salient distractors and the conditions that facilitate *clump scanning*. The present commentary traces the development of Theeuwes’ theorizing and carves out remaining discrepancies, most notably the conjecture of two qualitatively distinct search modes. Such a dichotomy is embraced by us, but decidedly rejected by Theeuwes. Accordingly, we selectively review some evidence in favor of search modes that appear crucial to the current debate.

Theeuwes’ persistent emphasizing of automatic capture by salient distractors (Theeuwes, 2010) and his invention of the additional-singleton paradigm (Theeuwes, 1991) were pivotal influences on our research. To provide sufficient context for our commentary on Theeuwes (2023)’s current theoretical stance, we briefly introduce ours. Liesefeld and Müller (2020) proposed two qualitatively different search modes, termed *priority guidance* and *clump scanning*, respectively: either focal selective attention is guided towards promising target locations via a *priority map* reflecting top-down (goals and experiences) weighted bottom-up salience signals (Found & Müller, 1996; Liesefeld & Müller, 2019), or observers scan one clump of stimuli after the other, comparing each stimulus against a *search template*. While clump scanning proceeds serially (typically in a systematic, though idiosyncratic manner), all stimuli within a clump (i.e., those encompassed within the attentional window) are processed in parallel. From the existence of these two search modes, it follows that a peak on the priority map produced by a salient distractor would affect search only if observers operate in priority-guidance mode, which relies on the priority map; and they would operate in this mode only if the map is useful to find the target. Because a non-salient target cannot produce a peak on the priority map (any weighting of zero salience will still result in zero priority), observers will tend to employ clump scanning instead of priority guidance to the degree that the target is non-salient.

In response to a recent target article co-authored by Theeuwes (Luck et al., 2021), we therefore devoted our entire commentary (Liesefeld et al., 2021) to the crucial, but largely overlooked, role that expected *target* salience plays for interference by a salient *distractor* (see Liesefeld et al., 2023, for definitions of terms). Accordingly, we consider the clarity with which Theeuwes (2023) now also embraces the relevance of target salience for the attentional-capture debate a major advance in his theorizing. While Theeuwes (2004) had equally stressed the relevance of target and distractor salience, Wang and Theeuwes (2020) placed much greater emphasis on distractor salience. In fact, their most influential claim was that the *distractor* in the Gaspelin et al. (2015) design is not salient – which we argued is not true and misses the point (Liesefeld et al., 2021; see also Liesefeld & Müller, 2019). So, encouragingly, this claim is no longer made in the present target article.

Likewise, the notion of clump-wise serial search takes center stage in the target article, whereas it was peripheral in Theeuwes (2004) and completely absent from the recent Wang and Theeuwes (2020) and Luck et al. (2021) papers – re-emerging only in response to our commentary (Theeuwes, 2021). We appreciate this development, because we did postulate parallel processing within clumps and the “boundary conditions” that facilitate it – notably, search displays with few and/or heterogenous nontargets – right from when we started discussing Gaspelin’s intriguing findings (Liesefeld & Müller, 2019, 2020).

While the above two points are clear, another aspect of Theeuwes’ (2023) theoretical position requires further explication. Other theorists had explained pop-out of salient targets among homogenous nontargets in two opposing ways: (a) attention is guided towards the target position to the degree that this position is highlighted on a priority map, with salience being the major bottom-up influence on priority/guidance (Wolfe, 1994; 2021); or (b) strong target-nontarget differences and nontarget-nontarget similarities allow the establishment of a search template that renders it efficient to reject nontargets as a group (Duncan & Humphreys, 1989; Humphreys & Müller, 1993). Theeuwes seems to assume that both (largely redundant) sets of mechanisms are at play concurrently. Mixing up the two distinct classes of theory might be owing to a confusion of *salience* and *discriminability*. The two are different (relative) features of a stimulus (Liesefeld et al. 2023; see Zhaoping, 2008, for a particularly impressive example): salience depends on the similarity of (close-by) physical stimuli (Nothdurft, 1992), whereas discriminability depends on the similarity of a physical stimulus and a mental representation (the *search template*; Bundesen, 1990; Duncan & Humphreys, 1989). In our theory, salience plays a major role in one search mode (priority guidance), whereas discriminability plays a major role in the other mode (clump scanning; Liesefeld et al., 2021; Liesefeld & Müller, 2020). In the latter, which is employed to find non-salient targets, there is, by definition, no need to “avoid” or “resist” capture by salient distractors, because salience simply plays no role in this mode, and salient distractors are not usually similar to the search template.

In contrast to our search-mode dichotomy, Theeuwes (2023) conceives of clump-wise processing to involve a (perhaps strategic) reduction in the size of the attentional window, in contrast to a display-encompassing window within which attention is guided. If these are not construed as qualitatively different search modes (the major deviation from our position), attention must also be guided within a clump. This is a theoretical possibility, but we must also ask: what determines which clump the reduced-size window is allocated to next? Since Theeuwes (2023) assumes that salient distractors are not processed, this allocation must somehow be informed. Consequently, he must assume two hierarchical levels of guidance: guidance *of* the window and guidance *within* the window – rendering the theory less parsimonious than it might appear at first (see Müller et al., 2017). In our clump-scanning conception, while guidance of the attentional window is conceivable in principle, it is not needed to explain distractor benefits. We assume that observers move the window in a spatially systematic, but often unguided and idiosyncratic way (e.g., an observer might consistently search a circular display in clockwise direction starting at the 1 o’clock position). A clump containing a salient distractor is, on average, rejected more rapidly than a clump of the same size containing exclusively non-salient distractors, because the salient distractor is more easily discriminable from the target.

To us, the most convincing proof of the existence of search modes is provided by Leber and Egeth (2006; see also Zehetleitner et al., 2012). Theeuwes’ (2023) theory can explain the behavior of the “feature-search” group in their test phase by assuming that participants adopted a smaller attentional window, which is why they were not (significantly) affected by distractor presence albeit performing generally slower. However, it is hard to see how this account would concurrently explain the observed perfectly flat (or even slightly negative) search slopes. With a smaller attentional window, it should take longer to process larger set sizes, resulting in positive slopes. We find it most probable that the “feature-search” group processed all stimuli in parallel as a single, display-encompassing clump (see “clump scanning (efficient)” in Fig. 1 of Liesefeld & Müller, 2020), and that this was a viable strategy because of the high template–nontarget discriminability.

Finally, we agree that Theeuwes’ (2004) Experiment 1 is a crucial demonstration of distraction during salience-guided search for a non-singleton target. However, across 7 failed attempts to replicate this experiment, Wienrich and Janczyk (2011) observed efficient search *without* distraction by a salient distractor. These studies can be reconciled by assuming that Wienrich and Janczyk’s participants adopted clump scanning (with a display-encompassing clump) whereas Theeuwes’ participants adopted priority guidance, just as Leber and Egeth’s participants adopted different modes for solving the exact same task. The task created by Theeuwes (2004) can apparently be solved with comparable efficiency by either search mode, and, as Liesefeld and Müller (2020) surmised, seemingly auxiliary details (e.g., in the wording of instructions) might tip the scale in favor of using one or the other mode.
